# The Emerging Role of Targeted Therapy and Immunotherapy in the Management of Brain Metastases in Non-Small Cell Lung Cancer

**DOI:** 10.3389/fonc.2017.00033

**Published:** 2017-04-05

**Authors:** Annick Wong

**Affiliations:** ^1^Medical Oncology, McGill University Health Centre, Montreal, QC, Canada; ^2^Medical Oncology, Hôpital du Suroît, Valleyfield, QC, Canada

**Keywords:** brain metastases, non-small cell lung cancer, targeted therapies, immunotherapy, intracranial responses

## Abstract

Lung cancer is the worldwide leading cause of cancer-related mortality in men and second leading in women. Brain metastases (BM) account for 10% of non-small cell lung cancer (NSCLC) patients at initial presentation, with another 25–40% developing BM during the course of their disease. In the last decade, the field of precision oncology has led to the discovery of a multitude of heterogenous molecular abnormalities within NSCLC as well as the development of tyrosine kinase inhibitors that target them. In this review, the focus will be on targeted therapy and immunotherapy that show efficacy in BM rather than conventional treatment for multiple BM (such as surgical resection, WBRT, or stereotactic radiosurgery).

## Introduction

Lung cancer is the worldwide leading cause of cancer-related mortality in men and second leading in women (1). Brain metastases (BM) account for 10% of non-small cell lung cancer (NSCLC) patients at initial presentation (2), with another 25–40% developing BM during the course of their disease (3). The general metastatic NSCLC population survival is approximately 12 months, with a median progression-free survival (PFS) range from 3 to 6 months (4). BM are associated with poor prognosis, and the median survival ranges from 2.4 to 4.8 months for patients with BM who receive whole-brain radiation therapy (WBRT) (5). While the standard of care for BM remains radiotherapy, determining the optimal treatment between high-dose-focused radiations via stereotactic radiosurgery (SRS) alone versus WBRT remains controversial. A retrospective multi-institutional retrospective study showed a survival advantage in patients with fewer than four BM less than 4 cm in size (*n* = 189 for NSCLC) who were treated with SRS compared to those treated with WBRT [adjusted hazard ratio (HR) for NSCLC, 0.58; 95% confidence interval (CI), 0.38–0.87; *P* = 0.01] (6).

The treatment of BM is important in maintaining a good quality of life and limiting cognitive impairment and neurological dysfunction. In the last decade, the field of precision oncology has led to the discovery of a multitude of heterogenous molecular abnormalities within NSCLC as well as the development of tyrosine kinase inhibitors (TKIs) that target them (7).

Patients with untreated BM have been excluded from most clinical trials of systemic therapy for two reasons: (1) historically poor prognosis and (2) presumed poor blood–brain barrier (BBB) penetration by experimental drugs. Thus, the efficacy of these drugs in controlling NSCLC-related BM remains controversial.

In this review, the focus will be on targeted therapy and immunotherapy that show efficacy in BM rather than conventional treatment for multiple BM (such as surgical resection, WBRT, or SRS), or some combination of the three.

## Anaplastic Lymphoma Kinase (*ALK*)-Rearranged NSCLC

The control and prevention of BM have emerged as an important therapeutic issue as systemic therapies with TKIs continue to improve the duration of disease control for patients with oncogene-driven NSCLCs (8). BM have been reported in about 24% of *ALK*-rearranged NSCLC patients at diagnosis, making intracranial activity an important feature of all *ALK*-targeted therapies (9).

### Crizotinib

Crizotinib was the first *ALK* and ROS1 (c-ros oncogene 1) inhibitor, approved for treatment of *ALK*-rearranged NSCLC. Crizotinib has shown evidence of potential clinical benefit in patients with a baseline of BM. PROFILE 1014, a phase 3 prospective study in *ALK*-positive NSCLC, demonstrated higher intracranial disease control rate (IDCR) with first-line crizotinib compared to chemotherapy in patients with treated BM. Although intracranial time to progression was improved, it was not significant (ITT population: HR, 0.60; 95% CI, 0.34–1.05; *P* = 0.069; treated BM present: HR, 0.45; 95% CI, 0.19–1.07; *P* = 0.063; BM absent: HR, 0.69; 95% CI, 0.33–1.45; *P* = 0.323) (10). In patients with BM, the PFS was greatly improved with crizotinib versus chemotherapy (BM present: HR, 0.40; *P* < 0.001; median, 9.0 versus 4.0 months, respectively) and in the intent-to-treat population (HR, 0.45; *P* < 0.001; median, 10.9 versus 7.0 months, respectively). IDCR in patients with BM was significantly higher with crizotinib compared with chemotherapy (56 versus 25% at 24 weeks, respectively) (10) (Table 1).

**Table 1:** **Intracranial effect of tyrosine kinase inhibitors ALK inhibitors and epidermal growth factor receptor (EGFR) inhibitors in trials in non-small cell lung cancer (NSCLC)**.

Trial	Treatment	IDCR	ICRR
**ALK inhibitors**			
PROFILE 1014 (10)	Crizotinib	56% at 24 weeks	Not described
	PEM + CBDCA or CDDP	25% at 24 weeks	Not described
Pooled analysis of Ref. (11)	Crizotinib	56% at 12 weeks (previously untreated)	18% (previously untreated)
PROFILE 1005 (12)			
PROFILE 1007 (13)			
ASCEND-1 (14)	Ceritinib	65% (pretreated)	34.5%
		79% (naïve)	
ASCEND-2 (15)	Ceritinib	80%	45%
ASCEND-3 (16)	Ceritinib	76.9%	61.5%
NCT01449461 (17)	Brigatinib	83% (measurable)	50%
		85% (non-measurable)	31%
NP28673 (18)	Alectinib	85.3%	58.8% (measurable)
		84.5% (pretreated)	46.4% (non-measurable)
NP28673 and NP28761 (19)	Alectinib	90.0% (measurable BM)	64.0% (measurable BM)
		85.3% (measurable and/or non-measurable BM)	42.6% (measurable and/or non-measurable BM)
			35.8% (prior RT)
			58.5% (non-prior RT)
J-ALEX (20)	Alectinib	92.9%	85.4%
ALTA (21)	Brigatinib	88% (90 mg)	36% (90 mg)
		83% (180 mg)	67% (180 mg)
NCT01970865 (22)	Lorlatinib	Not described	44% (targetable and non-targetable)
			60% (targetable)
**EGFR inhibitors**			
Pooled analysis of published data	Erlotinib or gefitinib	75.7%	51.8%
Fan et al. (23)			
Retrospective analysis	Pulsatile high-dose weekly erlotinib	Not described	67%
Grommes et al. (24)			
LUX-Lung 3 and LUX-Lung 6 (25)	Afatinib	Not assessed	Not assessed
BLOOM (26)	Osimertinib	Not described	76% (33% LM improvement and 43% LM SD)
BLOOM (27)	AZD3759	Not described	52.4% (measurable)

*IDCR, intracranial disease control rate; ICRR, intracranial response rate; PEM, pemetrexed; CBDCA, carboplatin; CDDP, cisplatin; BM, brain metastases; RT, radiotherapy; LM, leptomeningeal metastases; SD, stable disease*.

Furthermore, a retrospective pooled analysis of single-arm phase 1 and 2 studies of crizotinib in advanced *ALK*-positive NSCLC, PROFILE 1007 (13) and 1005 (12), demonstrated a median overall survival (OS) of 29.6 months for 120 patients who were allowed to continue crizotinib beyond progressive disease (PD) because they continued to derive clinical benefit from it. Only 18% of previously untreated BM patients achieved an overall intracranial response rate (ICRR), with an IDCR of 56% (95% CI, 46–66%) at 12 weeks (11) (Table 1).

Inevitably, the brain is the most common site of PD after resistance to crizotinib because of inadequate central nervous system (CNS) penetration of the drug or the biological change in the tumor (28). Hence, progression of preexisting or development of new intracranial lesions in up to 70% of patients while receiving therapy was a common manifestation of acquired resistance to crizotinib (29). Most systemic cytotoxic chemotherapies and some TKIs seem to cross the intact BBB inefficiently (30).

Limited intracranial response of crizotinib might be related to lower concentrations of the drug in cerebrospinal fluid compared with the plasma concentration (0.616 versus 237 ng/mL, respectively, 5 h after administration of a 250 mg dose) (31).

### Ceritinib

Ceritinib is another *ALK* inhibitor approved for *ALK*-rearranged NSCLCs that have progressed on crizotinib.

*In vitro* studies have found that ceritinib has a 20-fold greater potency to inhibit *ALK* than crizotinib and a 12-fold greater potency than alectinib (32). Ceritinib was found to cross the intact BBB in rats with a brain-to-blood exposure ratio of approximately 15%, although no human data exists (33).

In the phase 1 ASCEND-1 study, ceritinib demonstrated activity in *ALK*-rearranged locally advanced or metastatic cancer NSCLC patients, including both *ALK*-naïve and *ALK*-pretreated patients who had progressed following multiple lines of chemotherapy. Thirty one percent of the *ALK* inhibitor-naïve patients and 60% of *ALK* inhibitor-pretreated patients had BM, respectively. There were 94 patients with retrospectively confirmed BM and at least one post-baseline imaging. IDCR was 79% (15 of 19) in *ALK* inhibitor-naïve patients and 65% (49 of 75) in *ALK* inhibitor-pretreated patients (14). Overall ICRR was 34.5% (34) (Table 1).

In the ASCEND-2 phase 2 study, ceritinib showed a durable response in *ALK*-rearranged NSCLC patients who progressed on chemotherapy and crizotinib, including patients with BM. Moreover, 20 of the 100 patients with baseline BM had active target lesions at baseline. The investigator-assessed overall ICRR was 45% (95% CI, 23.1–68.5%), while the IDCR was 80% (*n* = 20, 95% CI, 56.3–94.3) (15) (Table 1).

In the ASCEND-3 phase 2 study of ceritinib in *ALK*-inhibitor-naïve NSCLC, 40.3% (50/124 patients) presented with BM at baseline. Fifty-four percent (27/50 patients) had received prior radiotherapy to BM. Updated data from ESMO 2016 showed an ICRR of 61.5% (8/13) in patients with measurable BM at baseline and an IDCR of 76.9% (10/13) (16) (Table 1).

### Alectinib

Alectinib is another powerful *ALK* inhibitor that has shown activity in crizotinib-resistant patients. A phase 2 study in *ALK*-positive NSCLC patients observed an objective response rate of 48% (35).

A hurdle in treating BM is achieving a higher rate of drug concentration in the brain because of the BBB. In animal models, alectinib has a high brain-to-plasma ratio (0.63–0.94) and activity in intracranial tumor implantation models (36). Alectinib penetrates into the CNS (2.69 nmol/L) where it is able to exceed the *in vitro* concentration for *ALK* inhibition (1.9 nmol/L) (35, 37). Alectinib human studies show a 50% CNS distribution, but of a 12-fold lesser potency than ceritinib (33).

Unlike crizotinib and ceritinib, studies also suggest that alectinib is not a substrate of P-glycoprotein (P-gp), a key drug efflux pump typically expressed in the BBB (36), thus allowing for a higher rate of drug penetration through the BBB.

Pooled data analysis of NP28761 and NP28673, two single-arm phase 2 trials, evaluated the CNS effect of alectinib in pretreated *ALK*-rearranged NSCLC patients (19). NP28761 was limited to North America only (NCT01871805), while NP28673 was a global study (NCT01801111). One hundred thirty-six patients had baseline measurable BM (60% of the overall study populations). For patients with baseline measurable BM, the ICRR was 64.0% (95% CI, 49.2–77.1%) with 11 (22%) complete responses (CR) in the brain, the IDCR was 90.0% (95% CI, 78.2–96.7%), and the duration of response (DOR) was 10.8 months (95% CI, 7.6–14.1 months) (19) (Table 1). For patients with measurable and/or non-measurable baseline BM, the IDCR was 42.6% (95% CI, 34.2–51.4%), the IDCR was 85.3% (95% CI, 78.2–90.8%), and the median DOR was 11.1 months (95% CI, 10.3 months to not evaluable) (19). For patients with prior radiotherapy (*n* = 95), the ICRR was 35.8% (95% CI, 26.2–46.3%) and 58.5% (95% CI, 42.1–73.7%) for patients without prior radiotherapy (*n* = 41) (Table 1).

Updated intracranial response data on the 61/138 patients with baseline BM the global phase 2 NP28673 study was presented at ESMO 2016. In the measurable BM group (*n* = 34), the ICRR was 58.8% (95% CI, 40.7–75.4), while IDCR was 85.3% (95% CI, 68.9–95.1) and the median DOR was 11.1 months (Table 1). In the measurable and non-measurable group (*n* = 84), the ICRR was 46.4% (95% CI, 35.5–57.7), while IDCR was 84.5% (95% CI, 78–91.5) and median DOR was 11.2 months (18) (Table 1).

More recently, J-ALEX, a phase 3 study comparing alectinib and crizotinib in treatment naive patients in Japan, showed an ORR of 85.4% in the alectinib group versus 70.2% in the crizotinib group (20). In patients with BM, the HR for alectinib versus crizotinib was 0.08 (95% CI, 0.01–0.61). The J-ALEX trial enrolled 14 patients with asymptomatic BM in the alectinib arm. Only one of the patients with BM treated with alectinib had progressed by the time of data cut-off (IDCR of 92.9%) (20) (Table 1). Thus, reducing CNS progression in patients with *ALK*-positive NSCLC with alectinib could be achievable if alectinib is used in the first-line setting.

The Global ALEX trial is currently ongoing, also comparing alectinib versus crizotinib in first-line *ALK*-positive NSCLC but on a global scale. If the J-ALEX results are confirmed with this trial, alectinib could likely replace crizotinib as the standard first-line therapy for *ALK*-positive NSCLC in the future, especially those with BM.

### Brigatinib, Lorlatinib, and Others

Brigatinib is an *ALK* inhibitor with preclinical activity against rearranged *ALK* and clinically identified crizotinib-resistant mutants. NCT01449461, a phase 1/2 single-arm, open-label, multicenter study in patients with advanced malignancies is ongoing. In a *post hoc* independent radiological review of patients with baseline BM, 6/12 patients with lesions ≥10 mm had a brain response (≥30% decrease in sum of longest diameters of target lesions) and 8/26 patients with only non-measurable lesions had disappearance of all lesions. ICRR for brigatinib with measureable BM was 50% and the IDCR was 83% (17). In non-measurable BM, the ICRR was 31%, IDCR was 85%, median intracranial PFS was 97 weeks, and median duration of intracranial response 82 weeks (Table 1). In ALTA, a phase 2 trial of brigatinib, ORR in arm A (90 mg qd) was 46% while ORR in arm B (90 mg qd for 7 days followed by 180 mg qd) was 54%. Seventy-one percent (arm A) and 67% (arm B) had BM (21) (Table 1).

Since CNS progression is a common site of relapse in NSCLC *ALK*/*ROS1* mutation patients, lorlatinib was developed as a selective brain-penetrant *ALK*/*ROS1* TKI active against most known resistance mutations. The phase 1 portion of the ongoing phase 1/2 study NCT01970865 enrolled patients with *ALK*+ or *ROS1*+ NSCLC with or without BM and were treatment naïve or had disease progression after ≥1 TKIs. Preliminary results revealed an objective ICRR of 44% in targetable and non-targetable lesions and 60% in targetable lesion, respectively (22) (Table 1).

Additional second-generation *ALK* inhibitors shown to have efficacy in the brain include ASP3026, X396, and entrectinib (38).

## Epidermal Growth Factor Receptor (*EGFR*) TKIs

### First-Generation TKIs

Approximately 33% of patients with NSCLC harboring tumors with *EGFR*-TKI-sensitizing mutations develop BM during treatment (39). Evidence suggests that *EGFR* TKIs have some limited BBB penetration (40, 41). In a pooled analysis including 464 patients from 16 trials to study the efficacy of *EGFR* TKIs in NSCLC patients with activating *EGFR* mutations with BM showed that *EGFR* TKIs produce significant beneficial effects, with a pooled objective ICRR of 51.8%, IDCR of 75.7%, median PFS of 7.4 months, and OS of 11.9 months (23) (Table 1).

Although erlotinib is effective for *EGFR* mutant NSCLC, CNS penetration is limited at standard daily dosing. Concentrations in cerebrospinal fluid exceeding the half maximal inhibitory concentration for *EGFR* mutant lung cancer cells in patients with BM and leptomeningeal metastases (LM) that developed despite standard daily erlotinib or other *EGFR* TKIs were achieved with weekly intermittent “pulsatile” administration of high-dose (1,500 mg) erlotinib (24). ICRR was 67% (Table 1). Median time to CNS progression was 2.7 months (range, 0.8–14.5 months), and median OS was 12 months (range, 2.5 months–not reached) (24).

### Second-Generation TKI

In both LUX-Lung 3 and LUX-Lung 6 studies, there was a non-significant trend toward improved PFS with afatinib versus chemotherapy in patients with asymptomatic BM (LUX-Lung 3:11.1 versus 5.4 months, HR = 0.54, *P* = 0.1378; LUX-Lung 6:8.2 versus 4.7 months, HR = 0.47, *P* = 0.1060) (25). In combined analysis, PFS was significantly improved with afatinib versus with chemotherapy in patients with BM (8.2 versus 5.4 months; HR = 0.50; *P* = 0.0297) (25).

Afatinib significantly improved the ORR versus chemotherapy in patients with BM. For LUX-Lung 3, ORR for afatinib was 70.0% (95% CI, 45.7–88.1) versus chemotherapy 20.0% (95% CI, 4.3–48.1) in patients with BM. The LUX-Lung 3 DCR for afatinib was 95.0% (95% CI, 75.1–99.9) versus chemotherapy 80.0% (95% CI, 51.9–95.7) in patients with BM. In LUX-Lung 6, ORR for afatinib was 75.0% (95% CI, 55.1–89.3) versus chemotherapy 27.8% (95% CI, 9.7–53.5) in patients with BM. The LUX-Lung 6 DCR for afatinib was 89.3% (95% CI, 71.8–97.7) versus chemotherapy 72.2% (95% CI, 46.5–90.3). There was no significant difference in OS in patients with BM who were treated with afatinib or chemotherapy.

These findings perhaps demonstrate a clinical benefit of afatinib in *EGFR* mutation–positive patients with NSCLC and asymptomatic BM. However, the role of afatinib in active BM remains to be clarified since this was an exclusion criterion in this study. ICRRs were not assessed in this study (Table 1). Therefore, no direct conclusions can be made regarding afatinib’s ability to cross the BBB in concentrations sufficient to elicit CNS responses.

Despite limited evidence of *EGFR* TKIs providing benefit in a few patients with *EGFR* mutation-positive NSCLC with BM, a clinical need for novel *EGFR* TKIs with improved efficacy against BM still exists.

### Osimertinib in Leptomeningeal Disease

Leptomeningeal metastases are seen in 3–5% of NSCLC (42) and in 9% of *EGFR* mutation-positive patients (43). Osimertinib is an irreversible *EGFR* TKI that targets activating mutations (*EGFR*m) and resistance mutations (T790M). Osimertinib induced sustained tumor regression in an *EGFR*m PC9 mouse BM model. PET imaging showed higher levels of osimetinib levels in NHP and mouse models, in contrast to rociletinib and gefitinib (39).

In previous trials, osimertinib demonstrated robust systemic activity in patients with *EGFR*m NSCLC and BM and has shown CNS penetration with sustained tumor regression in BM (44). In the phase 1 BLOOM study, two third-generation *EGFR* TKIs—osimertinib and AZD3759—were studied in patients with *EGFR* mutation-positive advanced NSCLC (26). Neurological function improved from baseline in 24% (5/21) patients. Radiological improvements in LM were seen in 33% (7/21) patients, and 43% (9/21) had stable disease (SD) (Table 1). Clearance of tumor cells from the CSF occurred in two patients at two consecutive visits. Time on treatment suggests durable clinical benefit, with 15 patients remaining on treatment, 7 of whom have been on treatment for >9 months.

### AZD3759 in BM

AZD3759 is a reversible inhibitor of *EGFR*-activating mutations that was designed to achieve high exposure in the plasma and CNS. AZD3759 has high passive permeability (29.5 × 10^−6^ cm/s) and is not a substrate of the efflux transporters Pgp or BCRP at the BBB. *In vivo*, AZD3759 reached distribution equilibrium in rats, mice, and monkeys (*K*_puu,brain_ and *K*_puu,CSF_ > 0.5), suggesting BBB penetration (45). AZD3759 induced significant tumor regression and dramatically improved animal survival in the BM model (45).

The AZD3759 cohort of the BLOOM trial evaluated the safety, tolerability, and early efficacy of AZD3759 in 29 patients with advanced *EGFR* mutation-positive NSCLC and metastases, including LM (27). Patients with non-LM BM were required to have at least one measurable intracranial or extracranial lesion. Patients with LM had a diagnosis confirmed by positive CSF cytology. All patients received at least one prior line of *EGFR* TKI therapy and chemotherapy. In addition, 34% of patients underwent prior whole-brain radiotherapy.

AZD3759 demonstrated encouraging intracranial antitumor activity. Among 21 patients with measurable BM, 11 patients demonstrated tumor shrinkage in the target brain lesion at AZD3759 doses of ≥50 mg BID (Table 1). In this group, there were three confirmed partial responses (PR) and three unconfirmed PRs. Among 22 patients with measurable extracranial lesions, 8 experienced tumor shrinkage, with 1 unconfirmed partial response. At the time of data cutoff, 5 of 29 patients remained on treatment with AZD3759. The longest duration of treatment was 48 weeks.

### Beyond *EGFR* and *ALK*

Apart from *EGFR* mutation and *ALK* translocations other distinct molecular subtypes of NSCLC depend on oncogenic molecular aberrations (driver mutations) for their malignant phenotype. Limited but promising data exist for the treatment of BM on novel molecular targets such as *ROS1, BRAF, KRAS, HER2, c*-*MET, RET, PIK3CA, FGFR1*, and *DDR2* (46).

## Immunotherapy

### Nivolumab

Nivolumab, a human IgG4 anti-PD-1 monoclonal antibody is active in the second-line treatment of metastatic NSCLC after progression on a platinum-based chemotherapy. Experience in routine clinical practice may differ from that seen in a controlled clinical trial. In a randomized phase 3 trial (CheckMate 017), the effect of nivolumab was studied in patients with advanced squamous NSCLC and central CNS metastases in a real-world, expanded access program (EAP) in Italy (47). Three hundred seventy-one patients participated in the EAP at 96 centers in Italy. Thirty-seven of 371 (10%) patients had asymptomatic and controlled CNS metastases. The DCR was 49% among patients with CNS metastases, with CR in 1 patient, PR in 6 patients, SD in 11 patients, and PD in 19 patients (Table 2), while the ORR in patients with CNS metastases was 7/37 (19%) (Table 2). OS rate at 12 months was 35% for patients with CNS metastases and 39% for all patients. The median OS was 5.8 months (95% CI, 1.8–9.8) for patients with CNS metastases and 7.9 months (95% CI, 6.2–9.6) for all patients. The PFS rate at 12 months was 31% for patients with CNS metastases and 27% for all patients. The median PFS was 4.9 months (95% CI, 2.7–7.1) for patients with CNS metastases and 4.2 months (95% CI, 3.4–5.0) for all patients.

**Table 2:** **Effect of immunotherapy on BM in non-small cell lung cancer trials**.

Trial	Treatment	IDCR	ICRR (%)
CheckMate 017 (47)	Nivolumab	49%	19
CheckMate 012 (48)	Nivolumab	Not described	16.7
NCT12085070 (49)	Pembrolizumab	Not described	33

*BM, brain metastases; IDCR, intracranial disease control rate; ICRR, intracranial response rate*.

In the Goldman et al. abstract 9,038 analysis presented ASCO 2016, pooled data from nivolumab studies [CheckMate 063 (50), CheckMate 017 (47), and CheckMate 057 (51)] were assessed to determine efficacy and safety of nivolumab in patients with previously treated, asymptomatic CNS metastases at baseline and patients with untreated, asymptomatic CNS metastases at baseline. The best response to most recent prior therapy demonstrated in the nivolumab with CNS metastases arm was CR/PR of 13/46 (28%), SD of 15/46 (33%), and PD of 18/46 (39%), compared to the docetaxel with CNS metastases arm with CR/PR 8/42 (19%), SD 13/42 (31%), and PD 18/42 (43%) (48). Among patients with pretreated CNS metastases, median OS was longer in the nivolumab group (8.4 months; 95% CI, 4.99–11.6) compared to the docetaxel group (6.2 months; 95% CI, 4.4–9.23). The frequency of and time to new CNS lesions were similar across treatment groups. Furthermore, 8/46 (17%) patients developed new CNS lesions in the nivolumab with CNS metastases arm with a median (range) of 3 (1.9–10.4) months, while 9/42 (21%) patients developed new CNS lesions in the docetaxel with CNS metastases arm with a median (range) of 2 (0.5–8.0).

Moreover, in CheckMate 012 Arm M, 2 of 12 patients (16.7%) with untreated CNS metastases achieved intracranial responses, including one intracranial CR lasting >10.5 months (48) (Table 2). These results support further investigation of nivolumab monotherapy in patients with NSCLC and asymptomatic CNS metastases.

### Pembrolizumab

Pembrolizumab, a fully human anti-PD-1 monoclonal antibody is approved in first- and second-line treatment of metastatic NSCLC. NCT02085070 is a phase 2 study of pembrolizumab in patients with metastatic melanoma and NSCLC with untreated or progressive BM. The effect of drugs on untreated BM remains unclear because most clinical trials exclude these patients. Early data demonstrated that there was an ICRR of 6/18 (33%) in the NSCLC on pembrolizumab 10 mg/kg arm, similar to the systemic response rate (49) (Table 2). The median OS was 7.7 months to date.

The available data for the use of anti-PD-1 agents in the treatment of BM do not yet include data on PD-L1 status. These data when available could suggest higher response rates based on the level of PD-L1 positivity.

## Conclusion

Current standard of care for BM that require immediate local intervention (based on symptoms, location, size, or other concerning features) is craniotomy with resection or radiation therapy. There is still a role in integrating locally ablative therapy (LAT) in combination with targeted therapy and immunotherapy in patients with oligometastatic BM that are limited or have low metastatic tumor burden (52).

Prior to the advent of second-generation therapies for BM developing while on crizotinib, the only alternatives were ablation of oligometastatic brain lesion with LAT and continuing crizotinib (28). Using WBRT with concurrent erlotinib (53) was also a viable option rather than changing to traditional chemotherapy.

However, recent data showing dramatic and prolonged responses in BM patients treated with *EGFR* and *ALK* TKIs have suggested that delaying LAT and WBRT may be a valid treatment option for patients with asymptomatic BM from NSCLC, especially for those with *EGFR*-activating mutations or harboring *ALK* rearrangement.

The challenge will be to determine the optimal sequence of agents and modalities (WBRT and SRS). Perhaps serial genotyping, the degree of BM symptoms, and the toxicity profiles will serve to individualize treatments and determine the role of these targeted therapies in the therapeutic armamentarium of BM.

## Author Contributions

The author confirms being the sole contributor of this work and approved it for publication.

## Conflict of Interest Statement

The author declares that the research was conducted in the absence of any commercial or financial relationships that could be construed as a potential conflict of interest.
